# A Rare Case of Chronic Lymphocytic Leukemia Transforming Into Waldenström Macroglobulinemia During Ibrutinib Therapy

**DOI:** 10.7759/cureus.75274

**Published:** 2024-12-07

**Authors:** Kazi Samsuddoha, Sylvester Homsy, Mohan Preet, Kamrun Naher

**Affiliations:** 1 Internal Medicine, State University of New York Downstate Medical Center, Brooklyn, USA; 2 Hematology and Oncology, State University of New York Downstate Medical Center, Brooklyn, USA; 3 Endocrinology, State University of New York Downstate Medical Center, Brooklyn, USA

**Keywords:** b-cell chronic lymphocytic leukemia (b-cll), ibrutinib therapy, lpl, lymphoplasmacytic lymphoma, waldenstrom macroglobinemia

## Abstract

Chronic lymphocytic leukemia (CLL) can rarely transform into Waldenström macroglobulinemia (WM), posing diagnostic and therapeutic challenges. The diagnosis of WM requires bone marrow infiltration by lymphoplasmacytic cells and the presence of IgM gammopathy. Immunophenotypic markers include FMC7+, CD19+, CD20+, and CD138+. The MYD88 mutation is characteristic. Symptoms arise from tumor infiltration and monoclonal protein production. Here, we present a case of CLL transforming into WM during treatment with ibrutinib. Given the rarity of such a transformation, this case may serve as a valuable reference, and further investigation is needed to understand the pathology underlying this transformation.

## Introduction

Chronic lymphocytic leukemia (CLL) is a chronic monoclonal neoplastic process characterized by the progressive accumulation of mature-appearing yet dysfunctional lymphocytes in the blood, bone marrow (BM), lymph nodes, and spleen. While CLL typically transforms into more aggressive lymphomas such as diffuse large B-cell lymphoma (DLBCL) or Hodgkin lymphoma, known as Richter's transformation, transformation into other forms of malignancies, like lymphoplasmacytic lymphoma (LPL) or Waldenström macroglobulinemia (WM), is rarer but does occur, posing diagnostic and therapeutic challenges. The diagnosis of WM requires findings consistent with LPL in the BM and the presence of IgM gammopathy in the peripheral blood [1]. BM biopsy sample typically shows at least 10% infiltration by small lymphocytes with lymphoplasmacytic features [2]. These infiltrates express FMC7+, CD19+, CD20+, CD22+, CD 5-/+, and CD23-, among others. The plasmacytic components express CD138+, CD38+, and CD 45- or dim. In the case of CLL, abnormal B-cells typically express FMC7-, CD5+, and CD23+. The MYD88 mutation is a consistent feature in LPL [3]. Symptoms of WM primarily arise from tumor infiltration and monoclonal protein production, leading to conditions such as cytopenias and hyperviscosity syndrome. Patients initially present with nonspecific symptoms, and incidental laboratory findings are crucial to establish diagnosis. Here, we present a case of CLL transforming into WM during ibrutinib therapy.

## Case presentation

Our patient is a 50-year-old man with a medical history that includes human immunodeficiency virus (HIV), for which he is on highly active antiretroviral therapy (HAART), hypertension, treated hepatitis C virus, treated neurosyphilis, and anal atypical squamous cells of undetermined significance (ASCUS). He has been regularly followed in the hematology clinic after being referred from the HIV clinic in 2017 for persistent lymphocytosis. Reflex peripheral flow cytometry showed small-to-intermediate monotypic B-cells with the following markers: CD45+, CD5+, CD10-, CD19+, CD20+, CD22+, CD23+, CD38-, FMC7+, HLA-DR+, and surface immunoglobulin (sIg) kappa+. The phenotype of this monoclonal B-cell lymphocytosis (MBL) was atypical/variant for CLL due to the expression of FMC7, leading to a diagnosis of CLL. 

The patient was initially asymptomatic, with a Rai stage of 0 and only peripheral lymphocytosis. Fluorescence in situ hybridization (FISH) analysis of his specimen using DNA probes for CLL revealed a deletion of the TP53 gene in 10.0% (cutoff, 6.8%) of nuclei. The patient progressed through the Rai stages as he developed splenomegaly and worsening anemia. As a result, ibrutinib therapy was initiated in March 2019, which improved his anemia, though he developed leukocytosis, as expected, at the onset of treatment. 

The patient later presented to the emergency department (ED) in June 2023 with symptomatic anemia requiring transfusions. His hospital course was complicated by intermittent fevers, for which broad-spectrum antibiotics were initiated. He continued to require multiple packed red blood cell (PRBC) transfusions intermittently due to severe anemia. He also developed scleral icterus, elevated total bilirubin, and hematuria. Although his lactate dehydrogenase (LDH), haptoglobin, and reticulocyte counts were normal, his IgM was more than 5000. Infectious workup, including imaging studies, was unremarkable but revealed splenomegaly, cholelithiasis, and mild mesenteric lymphadenopathy. Ultrasound (US), magnetic resonance cholangiopancreatography (MRCP), and hepatobiliary iminodiacetic acid (HIDA) scans showed no conclusive pathology, contributing to the current clinical scenario. The total bilirubin eventually trended downward, and WM was considered the primary differential at this point. 

The patient was started on dexamethasone and an increased dose of allopurinol for possible tumor lysis syndrome due to elevated LDH and uric acid levels. His serum viscosity was high at 7 cP, and plasmapheresis was initiated via a right internal jugular (IJ) Shiley catheter. Finally, the patient underwent a BM biopsy, which revealed hypercellularity for his age (70%) with infiltration of small lymphocytes and plasma cells. Lymphoplasmacytic infiltrate occupied nearly 50% of the BM. Myeloid lineage cells were present and showed appropriate maturation. Flow cytometry of the peripheral blood identified a monotypic B-cell population that was kappa-restricted and negative for CD5, CD10, and CD103. The patient had an IgM kappa M spike. Polymerase chain reaction (PCR) analysis of the BM showed a MYD88 mutation, confirming the diagnosis of WM.

## Discussion

WM is a rare disorder characterized by LPL in the BM along with IgM monoclonal gammopathy in the blood. Studies suggest that autoimmune diseases, chronic immune system stimulation, and hepatitis C virus infection are associated with WM. To diagnose WM, two criteria must be met: (1) IgM monoclonal gammopathy in the serum and (2) a BM biopsy sample showing at least 10% infiltration by small lymphocytes with plasma cell or plasmacytoid differentiation [1]. According to the International Myeloma Working Group, less than 10% BM infiltration is classified as IgM MGUS [2]. LPL tumors can also produce immunoglobulins, gamma heavy chains, or mixed cryoglobulins. In LPL, the BM infiltrate typically consists of varying amounts of plasma cells, small lymphocytes, and plasmacytoid cells. Cytoplasmic Russell bodies or pseudonuclear Dutcher bodies (periodic acid Schiff (PAS)-positive inclusion bodies) can form due to the accumulation of cytoplasmic IgM in some cells. Immunophenotypically, these infiltrates express CD5-/+, CD103-, CD23-, and FMC7+, whereas abnormal B-cells in CLL typically express CD5+, CD23+, and FMC7-. Genetic mutations play a central role in the pathogenesis of WM. In the malignant B-cells of WM, both chromosomal abnormalities and somatic mutations have been found. The most common mutation in LPL is MYD88 L265P, an activating point mutation [3,4], which supports the diagnosis but is not entirely specific. In WM, recurrent mutations in the CXCR4 gene have been identified in about one-third of patients [5]. Additionally, mutations in ARIDIA and CD79B have been found in LPL, though less frequently [6]. Somatic mutations largely influence clinical presentations and survival rates. In more than half of the BM-based cases, the most common chromosomal abnormality is the deletion of 6q21-q25, which is less common in lymph node-based diseases [7]. This deletion is associated with more pronounced symptoms, poor prognosis, and reduced survival [8]. Symptoms of WM are due to either tumor infiltration or monoclonal protein production. Tumor infiltration into hemopoietic tissues causes cytopenias, lymphadenopathy, and organomegaly. Central nervous system (CNS) infiltration can also occur. The abnormal monoclonal IgM can cause neuropathy as an autoantibody against myelin-associated glycoprotein, autoimmune cold hemolytic anemia when directed against its own RBC antigen, deposit in extracellular space of various organs as amorphous material often with a combination of amyloid, precipitate in cold manifesting symptoms of cryoglobulinemia. Hyperviscosity syndrome may develop, particularly when serum viscosity exceeds 4 cP. At presentation, patients often have nonspecific constitutional symptoms or may be asymptomatic, with incidental abnormal laboratory tests leading to the diagnosis. Elevated beta-2 microglobulin and anemia are common abnormalities at presentation [9]. Lymphadenopathy, hepatomegaly, and splenomegaly are observed in about one-fifth of LPL patients [10]. However, since BM findings are required for diagnosis, many LPL cases have reached stage IV disease at the time of diagnosis. In our case, a BM biopsy showed approximately 50% lymphoplasmacytic infiltration. Flow cytometry revealed a monotypic B-cell population that was negative for CD5, CD10, and CD103. The patient has an IgM kappa M spike and a MYD88 mutation, leading to the diagnosis of WM.

## Conclusions

Case reports on the transformation of CLL to WM are scarce. The exact cause of this transformation is not fully understood but may be influenced by autoimmune factors, chronic immune stimulation, and viral infections such as hepatitis C. The role of Bruton tyrosine kinase (BTK) inhibitors (e.g., ibrutinib) also warrants consideration, especially if genetic mutations lead to resistant clones. WM, characterized by lymphoplasmacytic lymphoma in the BM and IgM monoclonal gammopathy in the blood, presents a complex clinical picture, posing diagnostic and therapeutic dilemmas. Immunophenotypic markers and genetic mutations, such as MYD88 L265P and CXCR4, are important for diagnosis. Due to challenges in early detection, further research into genetic mutations and chromosomal abnormalities is essential.
